# SIV Infection Is Associated with Transient Acute-Phase Steatosis in Hepatocytes In Vivo

**DOI:** 10.3390/v16020296

**Published:** 2024-02-15

**Authors:** Nina Derby, Sreya Biswas, Sofiya Yusova, Cristina Luevano-Santos, Maria Cristina Pacheco, Kimberly A. Meyer, Brooke I. Johnson, Miranda Fischer, Katherine A. Fancher, Cole Fisher, Yohannes M. Abraham, Conor J. McMahon, Savannah S. Lutz, Jeremy V. Smedley, Benjamin J. Burwitz, Donald L. Sodora

**Affiliations:** 1 Center for Global Infectious Disease Research, Seattle Children’s Research Institute, Seattle, WA 98109, USA; 2 Vaccine and Gene Therapy Institute, Oregon Health and Science University, Beaverton, OR 97006, USA; 3 Oregon National Primate Research Center, Beaverton, OR 97006, USA; 4 Department of Laboratories, Seattle Children’s Hospital, Seattle, WA 98105, USA

**Keywords:** HIV, SIV, liver, hepatocyte, biopsy, steatosis, histology, metabolism

## Abstract

Metabolic-dysfunction-associated fatty liver disease (MAFLD) is a major cause of morbidity and mortality in HIV-infected individuals, even those receiving optimal antiretroviral therapy. Here, we utilized the SIV rhesus macaque model and advanced laparoscopic techniques for longitudinal collection of liver tissue to elucidate the timing of pathologic changes. The livers of both SIV-infected (N = 9) and SIV-naïve uninfected (N = 8) macaques were biopsied and evaluated at four time points (weeks −4, 2, 6, and 16–20 post-infection) and at necropsy (week 32). SIV DNA within the macaques’ livers varied by over 4 logs at necropsy, and liver SIV DNA significantly correlated with SIV RNA in the plasma throughout the study. Acute phase liver pathology (2 weeks post-infection) was characterized by evidence for fat accumulation (microvesicular steatosis), a transient elevation in both AST and cholesterol levels within the serum, and increased hepatic expression of the PPARA gene associated with cholesterol metabolism and beta oxidation. By contrast, the chronic phase of the SIV infection (32 weeks post-infection) was associated with sinusoidal dilatation, while steatosis resolved and concentrations of AST and cholesterol remained similar to those in uninfected macaques. These findings suggest differential liver pathologies associated with the acute and chronic phases of infection and the possibility that therapeutic interventions targeting metabolic function may benefit liver health in people newly diagnosed with HIV.

## 1. Introduction

The liver is the major metabolic and filtration organ in the body, receiving three quarters of its blood supply from the gastrointestinal tract through the portal circulation. In obese individuals, excessive nutrition promotes steatosis, the deposition of fat in the liver, and microbial dysbiosis which can also disrupt hepatic homeostasis [1,2]. Metabolic-dysfunction-associated fatty liver disease (MAFLD) begins when the amount of hepatocyte fat exceeds 5% and then progresses to an inflammatory condition. As the inflammation becomes more severe and collagen is deposited (fibrosis), the liver disease is termed metabolic-dysfunction-associated steatohepatitis (MASH). Advanced MASH is irreversible due to the chronic inflammation, cell death, and fibrotic scarring which compromise hepatocyte function [1,2]. Long term, MASH can lead to cirrhosis and/or hepatocellular carcinoma.

MAFLD/MASH can also result from other inflammatory conditions besides obesity and increasingly has become an issue for HIV-infected people on antiretroviral therapy (even when virally suppressed) [3,4,5,6,7,8,9,10,11]. Indeed, a recent U.S. multi-center multi-ethnicity study found steatosis in 49% of the HIV-mono-infected subjects sampled [12]. Little is definitively known about the liver-specific changes underlying HIV-related MAFLD, as collecting liver biopsies in people is challenging and often prohibitive. Here, we employed the SIV infection of rhesus macaques as a model to examine liver-associated pathologic changes. During SIV infection, immune changes and microbial alterations in the liver have been documented in numerous studies [13,14,15,16,17,18,19,20]. However, SIV infection studies to date have evaluated liver tissue obtained at just one time point, when the macaque is euthanized and necropsied. With the aim of obtaining information throughout the disease course, we utilized a laparoscopic technique [21,22] to acquire liver biopsies longitudinally throughout the first 32 weeks of the infection. We observed liver pathology as early as two weeks post-infection that evolved until the end of the study at 32 weeks post-infection. The early pathology included changes to aminotransferase and cholesterol levels in the blood as well as microvesicular steatosis in the liver, a type of hepatocyte fat generally associated with metabolic dysfunction [23]. Interestingly this pathology was transient and not detected at time points after 2 weeks post-SIV infection. Accompanying the steatosis during acute infection, we detected changes in the liver in the expression of genes involved in metabolism, particularly the peroxisome proliferator-activated receptor alpha (PPARalpha), which is involved in beta-oxidation and cholesterol metabolism. Chronic SIV infection (32 weeks) was generally associated with dilatation of the liver sinusoids especially near the central veins and a spatial evening of T cells throughout the lobule, indicating a disruption of efferent hepatic blood flow and immunological changes that persisted despite resolution of early metabolic disruption. These findings reveal the timing of pathogenic changes that occur within the liver during SIV infection.

## 2. Materials and Methods

### 2.1. Ethics Statement

The animal study was approved by the Institutional Animal Care and Use Committee (IACUC protocol # IP00001485) of the Oregon National Primate Research Center (ONPRC, Beaverton, OR, USA). All rhesus macaques involved in the study were managed according to the laws, regulations, and guidelines set forth by the United States Department of Agriculture, Institute for Laboratory Animal Research, Public Health Service, National Research Council, Centers for Disease Control, the Weatherall Report titled “*The use of nonhuman primates in research*”, and the Association for Assessment and Accreditation of Laboratory Animal Care (AAALAC) International. Nutritional plans used by the ONPRC consisted of standard monkey chow supplemented with a variety of fruits and vegetables as part of the environmental enrichment program established by the Behavioral Management Unit. Enrichment was distributed and overseen by veterinary staff with animals having access to more than one category of enrichment. SIV-infected macaques were kept in individual, adjoining cages allowing for social interactions with primate health observed daily by trained staff. All efforts were made to minimize suffering using minimally invasive procedures, anesthetics, and analgesics when deemed appropriate by veterinary staff. Animals were painlessly euthanized via sedation with ketamine hydrochloride injection followed by an intravenous barbiturate overdose following the recommendations of the panel of euthanasia of the American Veterinary Medical Association.

### 2.2. Study Design

A total of 17 adult Indian rhesus macaques were enrolled in the study. Nine macaques were infected intravenously with SIVmac251 at week 0. The other eight macaques served as SIV-naïve uninfected controls. Liver samples were obtained via laparoscopic biopsies using 3 mm biopsy forceps from each animal in the SIV group 4 weeks before SIV inoculation (week −4), as well as at 4 time points following infection: weeks 2, 6, 16–20, and necropsy (approximately week 32). Liver samples were collected from SIV-naïve macaques at the same times. Biopsies were approximately 3 mm × 2 mm × 2 mm in size and included the capsule. Blood samples were obtained at these and additional time points from all animals. Macaques were euthanized at the end of the experiment at 32 weeks post-SIV infection, or earlier if they met the end-point criteria for euthanasia established by the ONPRC. During the necropsy, the liver was removed intact and weighed. Liver tissue biopsies were washed in phosphate buffered saline (PBS) and either formalin-fixed and paraffin-embedded (FFPE) for microscopy or flash-frozen in liquid nitrogen and then stored at −80 °C for nucleic acid extraction. Blood collected in ethylene diamine tetra-acetic acid (EDTA)-coated tubes was separated via Ficoll Hypaque centrifugation to obtain plasma and peripheral blood mononuclear cells (PBMC). Clotted blood was centrifuged to obtain serum.

### 2.3. Nucleic Acid Extraction from Liver

Frozen liver tissue was placed in a lysing tube containing ceramic beads (Lysing Matrix D, MP Biomedicals, Santa Ana, CA, USA) with guanidine thiocyanate and phenol (Tri Reagent, Molecular Research Center, Cincinnati, OH, USA) and pulverized in a MagNA Lyser bead beater (Roche Life Science, Penzberg, Germany) at 6500 rpm for 45 s. The supernatant was then incubated with a 1/10 volume of bromochloropropane (Molecular Research Center) for 5 min and the organic and aqueous phases were separated via centrifugation at 12,000× *g* for 15 min at 4 °C. The RNA-containing aqueous phase was removed, and RNA was precipitated with isopropanol. RNA was then washed twice with 75% ethanol and air-dried before resuspending in 10 mM Tris, pH 8.0. The DNA-containing mixture of interphase and organic phase was incubated with a buffer of 4 M guanidine thiocyanate, 50 mM sodium citrate, and 1 M Tris and centrifuged at 12,000× *g* for 15 min at 4 °C. DNA was washed twice in 75% ethanol and air dried before resuspension with 10 mM Tris, pH 8.0.

### 2.4. SIV Quantification

Plasma SIV viral load was measured using a hybrid digital quantitative reverse transcriptase polymerase chain reaction (qRT-PCR) conducted by the Molecular Virology Core of the ONPRC. SIV DNA in the liver was measured via qPCR using SIV-specific primers and probe, with rhesus albumin primers and probe as a cell number control, according to the published protocol [24,25]. Reactions for each sample containing 500 ng DNA were run in duplicate on a Quant-Studio3 qPCR instrument using the following cycling conditions: 2 min at 56 °C for one cycle, 10 min at 95 °C for one cycle, and 15 s at 95 °C followed by one minute at 60 °C for 40 cycles.

### 2.5. Metabolic Profiling and Biomarker Analysis

HORIBA (Portland, OR, USA) was used to measure concentrations of the following analytes in serum at each time point at which liver samples were collected: total protein, albumin, alkaline phosphatase, alanine aminotransferase (ALT), aspartate aminotransferase (AST), gamma glutamyltransferase (GGT), total bilirubin, glucose, blood urea nitrogen, creatinine, potassium, sodium, chloride, magnesium, phosphorus, total cholesterol, and triglycerides. Plasma was also assayed for the fibrosis biomarker hyaluronic acid (HA) using a 1:20 dilution of samples acquired at weeks −4, 2, and necropsy and a commercial ELISA for monkey HA (Novatein Biosciences, Woburn, MA, USA). The assay was carried out according to the manufacturer’s instructions and read on a Spectramax i3× plate reader (Molecular Devices, San Jose, CA, USA).

### 2.6. Histology

Liver biopsies were fixed in 4% paraformaldehyde and paraffin embedded (FFPE) using the Integrated Pathology Core at the ONPRC. Tissue sections (5 mm) were placed on glass slides where they were deparaffinized in two washes of 100% xylene followed by rehydration in a graded ethanol series (100%, 95%, 70%, and 50%) before histological staining.

Hematoxylin and eosin (H&E) staining was carried out according to the manufacturer’s instructions (Electron Microscopy Sciences, Hatfield, PA, USA), and slides were mounted using Vector Hardmount media without DAPI (Thermo Fisher Scientific, Waltham, MA, USA). All sections contained at least ten lobules that could be identified by eye (clear central vein with surrounding portal triads). The H&E-stained slides were analyzed blindly by the study pathologist (MCP).

Collagen was detected in the liver tissues by staining slides with Picro Sirius Red Solution (Abcam, Waltham, MA, USA) according to the manufacturer’s instructions. Slides were mounted using Vector Hardmount media without DAPI, and brightfield images were obtained on the Keyence BZ-X-700 (Keyence Corporation of America, Seattle, WA, USA) microscope at 100–200× magnification. Upon importing the images into Fiji, a region of interest (ROI) was drawn just inside the boundary of the tissue section image to include all staining except that of the biopsy border/capsule. Red-stained collagen was identified via auto thresholding using the MaxEntropy method, and the auto threshold value was then reduced by 15% to prevent non-collagen tissue from being included in the analysis. The red-stained collagen was measured in proportion to the ROI size as a percentage, giving a collagen proportional area (CPA).

### 2.7. Hepatic Gene Expression Profiling

RNA extracted from macaque livers, as described above (Section 2.3), was converted to cDNA using the RT2 First Strand Kit (SABiosciences, Qiagen, Valencia, CA, USA) and subjected to transcriptional profiling for expression of genes involved in MAFLD using the RT2 profiler Human Fatty Liver PCR array (PAHS-157Z, Gene Globe, Qiagen). The Fatty Liver PCR array examines the expression of 84 hepatic genes involved in insulin and adipokine signaling, metabolism, inflammation, and apoptosis. Gene expression was analyzed using the RT2 Profiler PCR Array Data Analysis Template 2024 (Qiagen) via the 2^−∆∆Ct^ method. For each gene, the 2^−∆∆Ct^ for each animal was divided by the mean of the SIV-naïve macaques’ values for that gene, and this number was graphed as a fold-change from the mean of the SIV-naïve macaques.

### 2.8. Immunofluorescence Microscopy

Antibodies to CD3 (monoclonal rabbit anti-CD3 clone SP7, Thermofisher, Waltham, MA, USA, 1:150), arginosuccinate synthetase 1 (ASS1, monoclonal rabbit anti-ASS1 clone EPR12398, Abcam, 1:500), and glutamine synthetase (GS, monoclonal mouse anti-GS clone GT1055, Thermofisher, 1:500) were used to detect T cells and differentiate the periportal and centrilobular zones of the liver lobule, respectively. Tissue sections were deparaffinized and rehydrated as described for H&E staining, and antigen retrieval was performed using Antigen Unmasking Solution (Vector Laboratories, Newark, CA, USA) in a decloaking chamber at 90 °C for 30 min. After washing in TBS-T (0.025% TritonX-100 in Tris buffered saline) and blocking (0.1% bovine serum albumin and 1% goat serum in TBS-T), tissues were incubated with primary antibodies overnight at 4 °C, washed, and incubated with secondary antibodies for 60 min at room temperature protected from light. Secondary antibodies were goat anti-mouse-AlexaFluor488 (1:500) and goat anti-rabbit-AlexaFluor594 (1:500). Washed slides were mounted in Vectashield hard-set mounting medium with DAPI (Vector Labs) and imaged on a Keyence fluorescence microscope using a 20× objective lens to acquire a large section scan encompassing at least 6 lobules. CD3 staining was performed on one section and ASS1/GS staining on a consecutive section from the same tissue block. Using Fiji, regions of interest (ROIs) were manually drawn on the image from ASS1/GS staining to delineate the periportal (ASS1-bright, GS-negative) and centrilobular (ASS1-dim, GS-positive) zones. These ROIs were applied to the CD3-stained section and the T cells quantified within the ROIs by determining the area occupied by CD3 staining.

### 2.9. Statistics

All data obtained in the study except for the PCR array were analyzed using relevant non-parametric tests via GraphPad Prism version 9 software (San Diego, CA, USA). The number of macaques exhibiting a particular liver condition was evaluated between groups using the Fisher’s exact test. Periportal vs. centrilobular T cell location within a macaque liver section was compared using the Wilcoxon matched pairs and Mann–Whitney tests. Longitudinal data were analyzed using a mixed-effects model with Dunnett’s multiple comparison test. Correlations were established by identifying the Spearman correlation coefficient and by performing linear regression analysis. In all cases, results were deemed significant for *p* < 0.05. For PCR arrays, the data were analyzed within the RT2 Profiler PCR Array Data Analysis spreadsheet 2024. Results with *p* < 0.05 were considered significant, and results with *p* < 0.10 were graphed to indicate trends.

## 3. Results

**Figure 1:**
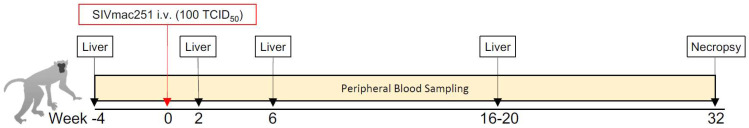
Study design and liver biopsy collection time points. Nine adult rhesus macaques were infected intravenously with 100 TCID50 SIVmac251 and followed for up to 32 weeks. Laparoscopic liver biopsies were collected at the indicated time points, and peripheral blood was collected throughout. Eight SIV-naïve (uninfected) macaques were bled and biopsied in parallel as controls.

**Figure 2:**
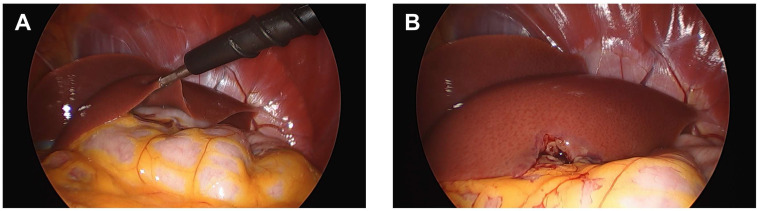
Laparoscopic liver biopsy procedure. Liver biopsies were collected as described in Zevin et al. [21]. (**A**) Forceps taking the biopsy. (**B**) Liver visualized immediately after retracting the forceps.

To assess the impact of SIV infection on liver histology and other functional readouts, we infected nine rhesus macaques intravenously with SIVmac251 and compared them to eight SIV-naïve, uninfected macaques. The timeline (Figure 1) indicates when liver biopsies were obtained, which includes 4 weeks prior to infection as well as 2, 6, 16–20, and 32 weeks post-infection or the time of necropsy. To obtain these longitudinal liver biopsies, we used a minimally invasive laparoscopic approach described previously [21]. Photos depict the macaque liver during and just following the completion of the procedure (Figure 2). To minimize bleeding at the biopsy site, additional pressure from the forceps was applied prior to the biopsy and afterwards when necessary. This technique was utilized throughout the study without any adverse issues.

**Figure 3:**
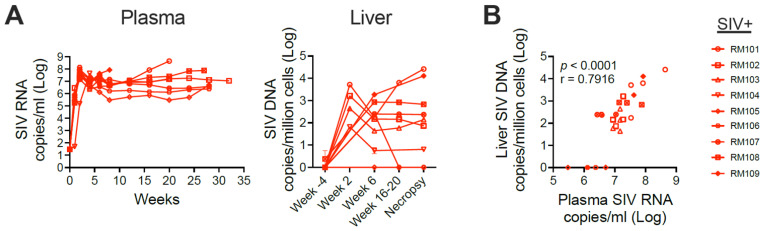
SIV replicates in the liver in rhesus macaques. (**A**) Plasma viral loads (left) and liver SIV DNA copies (right) were enumerated in SIV-infected macaques. (**B**) Spearman correlation analysis was performed to evaluate the relationship between plasma viral load and liver SIV DNA. Each symbol represents an animal’s plasma viral load and liver SIV DNA quantity at a given sampling time point.

Monitoring of SIV infection identified high acute and set-point SIV RNA plasma viral loads that were comparable to viral loads observed in other studies of SIVmac251 infection in adult rhesus macaques (Figure 3A, left) [26]. Within the liver, SIV DNA reached up to 5 × 10^3^ copies per million cells 2 weeks post-infection, the time of acute plasma viral load peak. In some of the macaques, even higher liver SIV DNA viral loads were observed at the time of necropsy; this was generally associated with a more rapid rate of disease progression (Figure 3A, right). In all animals across the time course, the levels of SIV DNA in the liver correlated strongly with levels of SIV RNA in the plasma (Spearman correlation *p* < 0.0001) (Figure 3B). One SIV-infected rhesus macaque (RM105) had no SIV DNA detectable in the liver at any time assayed post-infection (note that a liver sample was not available from 2 weeks post-infection for RM105).

**Figure 4:**
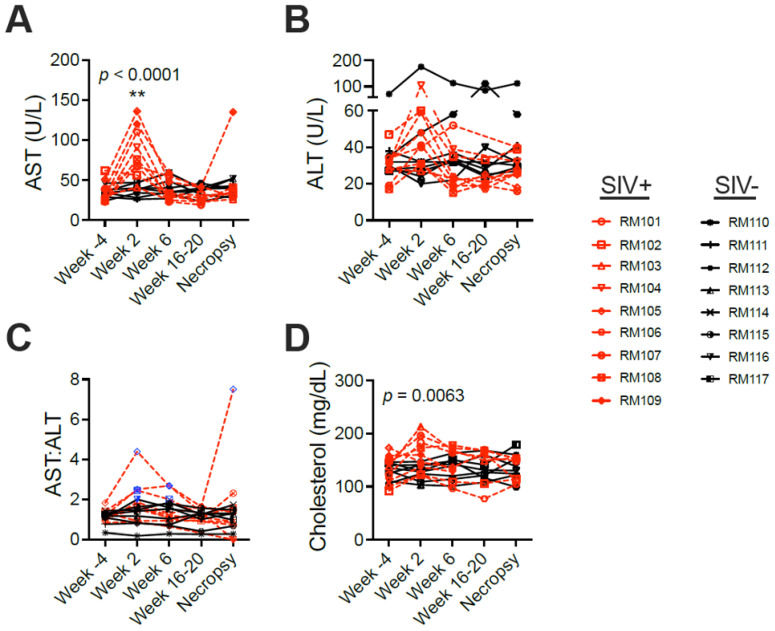
SIV infection results in metabolic changes during acute infection. Metabolic changes were monitored in SIV-infected (SIV+) and SIV-naïve (SIV−) macaques’ serum over time by assaying for sixteen analytes. Shown are the analytes that were different between SIV+ and SIV− groups: (**A**) Aspartate aminotransferase (AST); (**B**) Alanine aminostransferase (ALT); (**C**) The ratio of AST to ALT; (**D**) Total cholesterol. In (**C**), blue symbols denote values at or greater than 2.0. *p*-values reflect the Time × Treatment interaction of comparing the SIV+ and SIV− groups against each other over time in a mixed effects model. Asterisks indicate significant changes in metabolite concentrations at post-SIV times relative to week −4 within a group. ** shows *p* < 0.01.

We quantified concentrations of serum metabolic markers in the macaques throughout the disease course, including enzymes that are associated with liver disease in humans. Aspartate aminotransferase (AST) was significantly elevated in SIV-infected compared to SIV-naïve macaques when considering data from the entire time course (*p* < 0.0001) and specifically at the 2-week time point (*p* < 0.01) (Figure 4A, Supplementary Table S1). Alanine aminotransferase (ALT) was also elevated at week 2 in the SIV-infected macaques but was not significantly different between the groups due to a high concentration of ALT in one SIV-naïve macaque across all time points and an increase late in the study in a second macaque. (Figure 4B, Supplementary Table S1). Exclusion of the one macaque with consistently elevated ALT exposed a significant difference between the groups in ALT (*p* = 0.0018). The AST:ALT ratio has been used in previous studies as an indicator of alcoholic liver disease (ratios at or greater than 2) including in the macaque model [27] and has been shown to be elevated in MASH [28,29]. Here, the AST:ALT ratio was greater than 2 in three SIV-infected animals at two or more time points and in one SIV-infected animal at a single time point (Figure 4C). By comparison, this ratio was equal to 2 in only one SIV-naïve animal at one time point. Finally, total cholesterol was also elevated in the SIV-infected macaques over the course of the study (*p* = 0.0063), particularly at week 2. Interestingly, the week 2 increases observed in AST, ALT, and cholesterol resolved to baseline by 6 weeks post-infection and remained similar to those parameters in the SIV-naïve cohort for the remainder of the study (Figure 4D, Supplementary Table S1). In contrast, AST:ALT remained elevated at late study time points in SIV-infected macaques.

**Figure 5:**
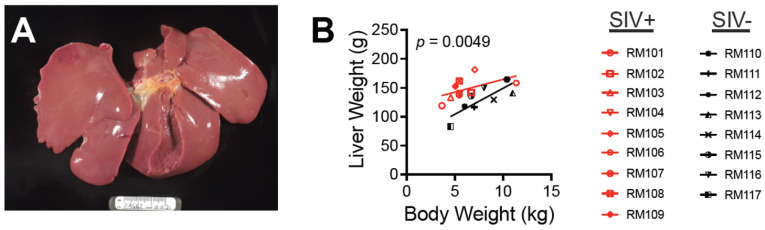
SIV infection unlinks liver weight from body weight during chronic infection. At necropsy, the liver was removed and liver weight and body weight were evaluated. (**A**) Intact liver after removal. (**B**) Spearman correlation analysis was performed to evaluate the relationship between liver weight and body weight. Linear regression lines for each group are shown. *p*-value reflects the comparison of the regression lines for SIV+ and SIV− groups.

To assess the relationship between the weight of the liver at 32 weeks and body weight, we carefully removed the intact livers at the time of necropsy (Figure 5A) and evaluated the weight with respect to total body weight (Figure 5B). There was no significant difference in either body weight or liver weight between the groups. However, the liver weight:body weight relationship was significantly different in SIV-infected compared with SIV-naïve macaques (*p* = 0.0049) (Figure 5B). While liver weight in SIV-naïve macaques correlated with body weight (r^2^ = 0.6472; *p* = 0.0161), no correlation was evident for the SIV-infected macaques (r^2^ = 0.2857; *p* = 0.1382) (Figure 5B). Interestingly, the three SIV-infected macaques with the highest liver weight were also those with two or more time points at which the AST:ALT ratio was elevated.

**Figure 6:**
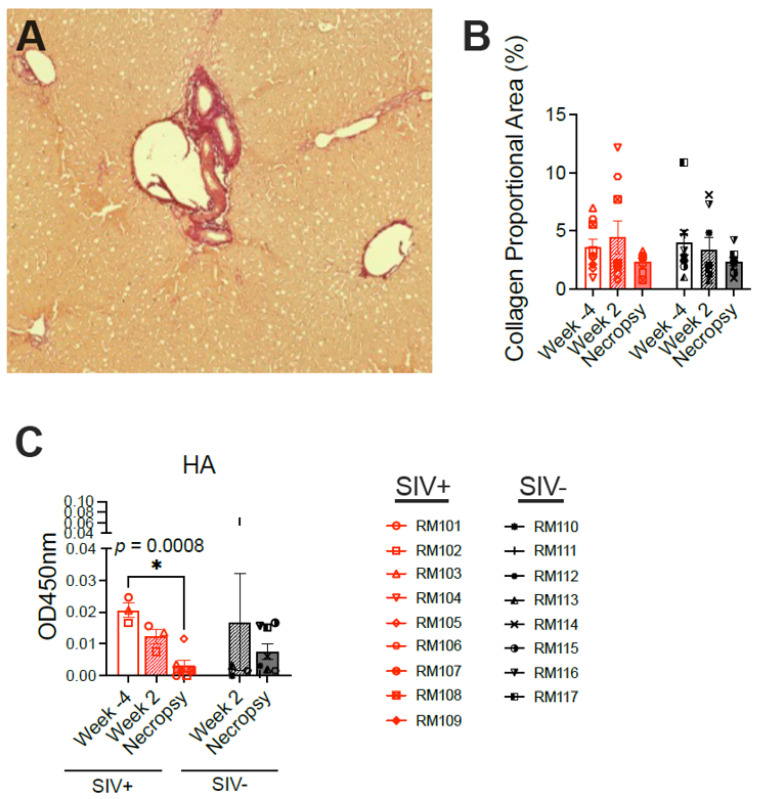
SIV infection in rhesus macaques does not cause significant fibrosis in the liver. (**A**) Sirius Red staining highlights collagen deposition in the liver; normal collagen in a portal triad is shown highlighted in red. (**B**) The area of the liver section occupied by collagen was measured before infection (week −4), during acute infection (week 2), and during chronic infection at necropsy. (**C**) Hyaluronic acid, a glycosaminoglycan component of the extracellular matrix and biomarker of fibrosis, was measured in the plasma via commercial ELISA using samples from weeks −4, 2, and necropsy (OD450 nm 0.07 corresponds to 125 pg/mL based on the standard curve).

To explore the type of liver pathology induced by SIV infection, liver tissue was first stained for collagen using Sirius Red (Figure 6A). Normal collagen as well as pathologic increases in collagen secondary to scarring are highlighted via Sirius Red staining. However, no significant difference in collagen deposition was observed between SIV-infected and SIV-naïve macaques at either the week 2 or necropsy time points nor were any changes evident over time in the SIV-infected macaques (Figure 6B). Hyaluronic acid (HA), a glycosaminoglycan component of the extracellular matrix, is used as a plasma biomarker of tissue fibrosis in humans and correlates with the degree of hepatic fibrosis in individuals with chronic hepatitis B virus infection [30]. The concentration of HA was previously observed to be elevated in the blood of rhesus macaques experiencing alcoholic liver disease [27]. Quantification of HA in the plasma of the macaques here revealed no difference between the SIV-infected and SIV-naïve macaques at either week 2 or necropsy (Figure 6C), supporting the absence of fibrosis detected using Sirius Red. While the concentrations of HA were very low in the plasma from all animals at all time points (OD450 nm < 0.07 corresponding to <125 pg/mL), there was significantly less HA detected in the SIV-infected macaques at necropsy compared with the week −4 baseline (Figure 6C).

**Figure 7:**
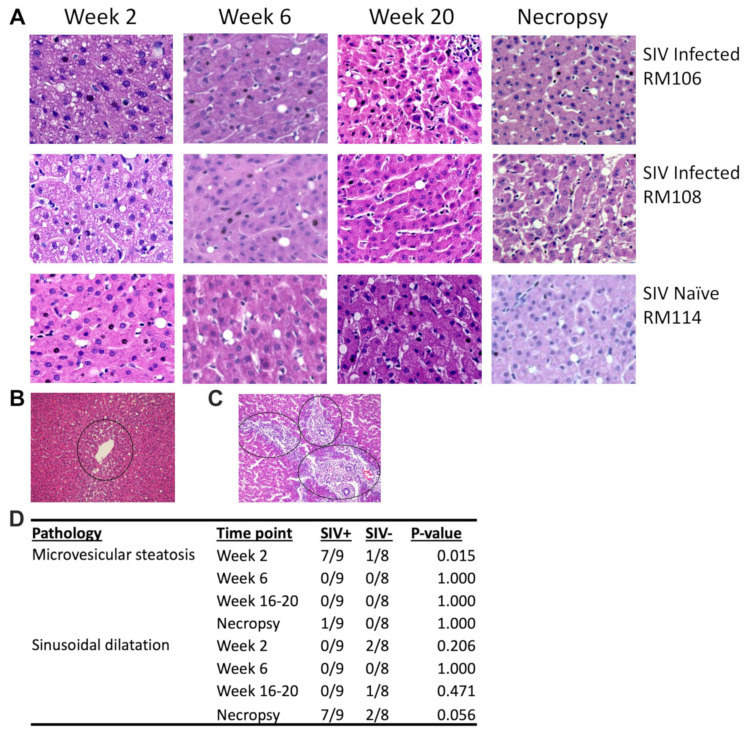
SIV infection results in histological changes consistent with liver dysfunction. H&E staining was performed on liver sections from SIV-infected and SIV-naïve macaques to evaluate liver pathology. (**A**) H&E-stained tissues are shown from two SIV-infected and one SIV-naïve macaque at weeks 2, 6, 20, and 32 (necropsy). Microvesicular steatosis is evident in the SIV-infected macaque livers at week 2 and sinusoidal dilatation can be seen at necropsy. (**B**) A zoomed-out image highlights the zonation of sinusoidal dilatation around a central vein at the necropsy time point of an SIV-infected macaque. (**C**) Portal inflammation was detected during chronic or advanced SIV infection in two macaques (necropsy time point). (**D**) Significant increases in liver pathology in the SIV-infected group were confirmed via a Fisher’s exact test.

Further blinded histological evaluation of the livers over the entire time course identified various mild pathologic findings in both macaque groups (Supplementary Table S2). One consistent observation was that adult macaque livers have a background level of approximately 5% macrovesicular steatosis. Individual macaques in both groups also exhibited a range of other mild histologic abnormalities including focal mild inflammation, venulitis, the occasional presence of iron, and apoptotic hepatocytes (Supplementary Table S2). However, there were also pathologic findings specifically associated with SIV infection. At the 2-week time point, microvesicular steatosis was detected in SIV-infected macaques more often than in SIV-naïve macaques (*p* = 0.015). Microvesicular steatosis was identified as small, non-nucleus-displacing fat droplets within hepatocytes, in contrast to the large, nucleus-displacing fat droplets that characterize macrovesicular steatosis (Figure 7A, Supplementary Table S2). This observation was only transient, as it was no longer significantly associated with SIV infection by 6 weeks post-infection nor any later time points (Figure 7A,D). Interestingly, the detection of microvesicular steatosis was coincident with the rise and fall in serum liver enzymes and cholesterol.

**Figure 8:**
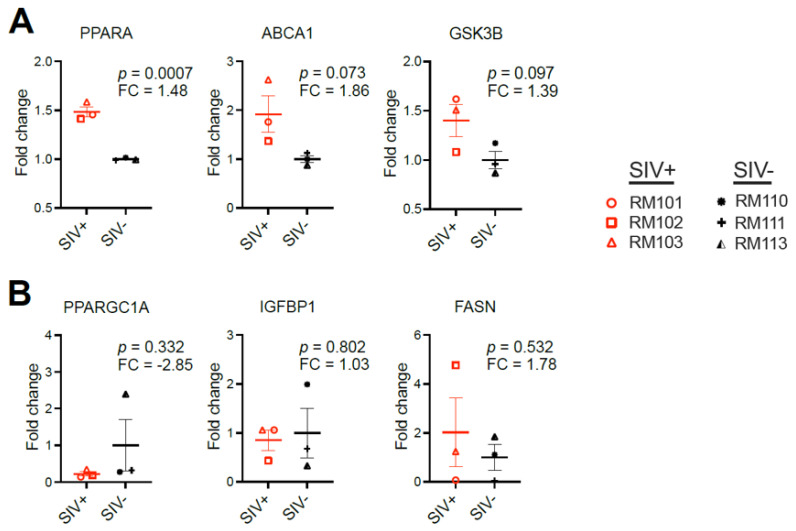
Acute SIV infection is associated with altered liver metabolism. Changes in the expression of 84 genes associated with steatotic liver disease were profiled using the RT2 PCR array for fatty liver disease using RNA extracted from 3 SIV-infected (SIV+) and 3 SIV-naïve (SIV−) macaques with liver samples available at the 2-week time point. (**A**) The 3 genes that were different (*p* < 0.10) between the SIV+ and SIV− macaques (PPARA, ABCA1, and GSK3B). (**B**) The 3 genes with the greatest fold-change in an alcoholic liver disease model in macaques are also depicted (PPARGC1A, IGFBP1, and FASN) [27].

Previous work in a rhesus macaque model of alcoholic liver disease identified changes in hepatic gene expression associated with steatosis and fibrosis in that model [27]. Exploring fatty-liver-associated transcriptional changes in the liver at the 2-week time point in six macaques from our cohort revealed a significantly higher expression of PPARA in the three SIV-infected vs. three SIV-naïve macaques (*p* = 0.0007) (Figure 8A). PPARA is a component of the cholesterol metabolism, beta oxidation, and adipokine signaling pathways. Trending but non-significant increases were also observed in other cholesterol and carbohydrate metabolism genes: ABCA1 (*p* = 0.073) and GSK3B (*p* = 0.097) (Figure 8A). The most upregulated genes associated with steatosis in the macaque alcohol liver disease model (PPARGC1A, IGFBP1, and FASN) [27] were not altered by acute SIV infection (Figure 8B).

Assessment of the macaques at necropsy identified a second pathologic finding, sinusoidal dilatation, which could be observed in some of the SIV-infected macaques (*p* = 0.056) (Figure 7B,D, Supplementary Table S2). Sinusoidal dilatation is defined as a blockade to the outflow of blood from the liver that is characterized by the presence of enlarged sinusoids, particularly around the central veins. In addition, two SIV-infected (and none of the SIV-naïve) macaques at necropsy exhibited moderate levels of inflammation and/or immune cell infiltration, particularly at the portal triads (Figure 7C). Of note, one of these was the macaque with no SIV DNA detected within the liver, suggesting that factors besides the virus may participate in liver pathology during SIV infection.

**Figure 9:**
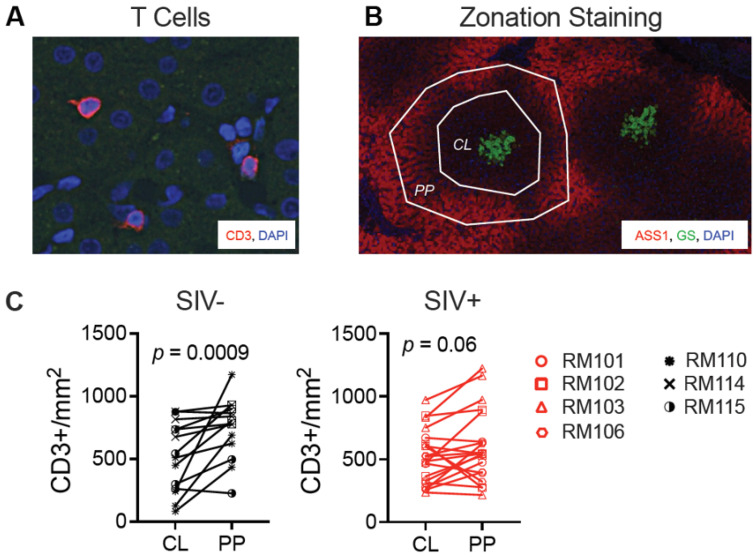
T cells shift towards the central vein in chronic SIV infection. Immunofluorescence microscopy was used to identify the location of T cells within the liver lobules. (**A**) Antibody to CD3 was used to mark T cells in liver sections. (**B**) Antibodies to arginosuccinate synthetase-1 (ASS1) and glutamine synthetase (GS) were used to identify the periportal (PP, ASS1-bright/GS-negative) and centrilobular (CL, ASS1-dim/GS-bright) zones of the liver lobule. (**C**) T cells were quantified in each lobule zone in a randomly selected subset of SIV-naïve (n = 3) and SIV-infected (n = 4) macaques. Five lobules were assessed from each macaque liver and the number of T cells in each zone was compared via a Wilcoxon matched-pairs test.

To further characterize the liver changes during chronic infection, we examined T cell locations via immunofluorescence microscopy. We previously reported that liver T cells are infected with SIV (RNA-scope positive) and observed primarily within the portal tracts [18]. We identified T cell locations via immunostaining for CD3 (Figure 9A) in a randomly selected subset of SIV-naïve (n = 3) and SIV-infected (n = 4) macaque livers at the time of necropsy. Two markers were used to define liver lobule zonation, arginosuccinate synthetase 1 (ASS1), and glutamine synthetase (GS). ASS1 is an enzyme produced by periportal hepatocytes marking the periportal zone (PP), while GS is produced by pericentral hepatocytes and marks the central vein and centrilobular (CL) zone of the lobule (Figure 9B). In SIV-naïve macaque livers, the T cells were skewed towards the periportal zone as significantly more T cells within a given lobule were present in the periportal (ASS1-bright, GS-negative) than in the centrilobular (ASS1-dim, GS-positive) zone (*p* = 0.0009) (Figure 9C). However, in SIV-infected macaques, the T cells were no longer primarily in the periportal region (*p* = 0.06), with some lobules exhibiting more T cells in the centrilobular than the periportal zone (Figure 9C). The loss of periportal spatial skewing was not due to overall loss or accumulation of T cells as there was no significant difference in the total number of T cells counted between the groups (*p* = 0.34). These data suggest a shift in hepatic T cell location during chronic SIV infection.

## 4. Discussion

Studies in humans have indicated that HIV infection negatively impacts liver function [3,12]. However, teasing out the specific pathologies and their relationship to the stage of infection and immunological changes in the absence of confounding factors such as antiretroviral drug use, co-infections (e.g., hepatitis viruses), and behaviors (e.g., alcohol, illicit drugs, and diet) has been challenging. Using the SIV macaque model and collection of longitudinal biopsies of the liver, we evaluated the timing of liver pathology throughout the infection, focusing here on SIV infection in the absence of antiretrovirals. Longitudinal sampling is critical to this given inter-animal variability, and the use of minimally invasive laparoscopic sampling permits this evaluation while minimizing impacts to the model and optimizing animal welfare. Moreover, the longitudinal sampling revealed patterns that were distinct from that which we predicted. Initially, we had hypothesized that we would observe an increase in specific pathologies with progression of the SIV disease. But, surprisingly, we observed a liver pathology during the acute time points post-SIV infection that was distinct from the pathology observed during the chronic stages of the infection.

At two weeks post-infection, we observed several changes that coincided with SIV peak viral load in the plasma and high levels of SIV DNA in the liver. This early time point coincided with an increase in serum concentrations of AST and cholesterol in plasma, along with microvesicular steatosis and increased expression of the cholesterol metabolism gene PPARA in the liver tissue. The increases in aminotransferase and cholesterol concentrations were consistent with those observed in published non-human primate studies of drug-induced diabetes [31], castration-induced metabolic change [32], and high fat diet [19]. Elevated circulating concentrations of enzymes associated with liver dysfunction are also seen in HIV-infected patients and indicated as a diagnostic for HIV infection [33,34,35], supporting the clinical relevance of the macaque findings.

Our results from SIV-infected macaques underscore the early damage inflicted on the liver by SIV/HIV infection itself and indicate a specific role for damage to the hepatocytes that release these enzymes. Although HIV does not infect hepatocytes, the virus can interact directly with hepatocytes through gp120 binding to the chemokine receptors used for virus entry (CCR5 or CXCR4) when these receptors are expressed on the hepatocyte surface [36]. CCR5 may also be expressed on hepatic stellate cells, and triggering of the stellate cells through this receptor may also result indirectly in hepatocyte damage [37]. Steatosis has also been shown to directly impair hepatocyte function [38], and the presence of the microvesicular form of steatosis in the macaques further points to hepatocyte damage during the acute SIV infection period. Unlike macrovesicular steatosis in which the hepatocyte is filled with a single large fat droplet that displaces the nucleus, microvesicular steatosis is caused by severe dysfunction of the mitochondrial beta-oxidation pathway and characterized by the presence of many small fat droplets that leave the nucleus in place and give the cytoplasm a foamy appearance [23]. Several conditions are indicated to cause microvesicular steatosis, including acute fatty liver in pregnancy, drug toxicity, and the combination of virus infection and drug (aspirin) in the case of Reye’s syndrome in children [23,39]. In people living with HIV, nucleoside reverse transcriptase inhibitors (NRTIs) have been shown to lower mitochondrial DNA levels and impair fatty acid oxidation, which can lead to microvesicular steatosis [40,41], while mitochondrial dysfunction was also documented within the adipose tissue of untreated HIV-infected patients [42]. And, finally, in a mouse model of lymphocytic choriomeningitis virus (LCMV) infection, microvesicular steatosis in hepatocytes was linked with a deficiency of CCR5, implicating a key HIV coreceptor in this process [43]. Here, microvesicular steatosis was present concurrent with the heightened expression of PPARA, a gene involved in cholesterol metabolism and beta-oxidation, underscoring the metabolic dysfunction in the liver during acute SIV infection.

The microvesicular steatosis and elevations in AST/cholesterol levels during acute SIV infection resolved in all animals, coincident with the decline in hepatic SIV DNA detection. Relatively little pathology was observed between weeks 2 to 20. At necropsy, we observed an increase in SIV DNA in some macaques; however, serum aminotransferases and microvesicular steatosis were not elevated, indicating that SIV infection itself may not be directly or solely responsible for this liver dysfunction. This is consistent with the observation that the macaque with no SIV DNA detected was also the one with the highest AST level in blood during acute infection as well as at necropsy. Interestingly, most SIV-infected macaques experienced sinusoidal dilatation in the liver at the 32-week time point. Enlargement of the sinusoids is generally due to blocked blood flow out of the liver or is related to vascular dysfunction [44,45]. The SIV-infected macaques exhibited the characteristic sinusoid enlargement near the central veins (at the center of the liver lobules), as this is where the blockage initiates. This type of sinusoidal lesion was described early in the HIV epidemic in autopsies of late-stage AIDS patients with documented liver dysfunction [46,47,48]. Another notable observation at the last time point (necropsy) was the dissociation of liver weight from body weight in the SIV-infected macaques. Wasting is a common occurrence in untreated chronic HIV and SIV infections and body weight loss due to the infection could have contributed to this dissociation. While it is possible that fibrosis could also impact liver weight, we did not observe fibrosis via Sirius Red staining nor did we find elevated HA in plasma. Notably, liver fibrosis was present in pig-tailed macaques infected with SIVsab for a similar amount of time and was more pronounced in the context of high-fat-diet feeding [19]. It is interesting to speculate that sinusoidal dilatation could also be a contributor to the increased liver weight per body weight in the SIV-infected macaques in our study as increased liver water weight was associated with sinusoidal dilatation in Leishmania infection of mice [49]. One potential explanation for the discrepancy in liver weight to body weight ratios is that the biopsies could have had differential outcomes in the SIV-infected versus SIV-naïve macaques, possibly due to differences in healing and fibrosis in the presence of the SIV infection. While we cannot rule this out, no evidence for differential healing in the two groups was noted by the pathologists.

The observation that T cells are predominantly periportal in SIV-naïve macaques is consistent with observations in mice that macrophages and natural killer cells reside in the periportal zone during homeostasis [48]. However, at the necropsy time point, T cells were spatially distributed more evenly between the periportal and centrilobular zones of the SIV-infected liver lobules. Published work indicates that SIV-infected cells within the liver during chronic infection are predominantly T cells in the portal tracts [18]. The fact that T cells shift towards the central veins late during SIV infection suggests the possibility that CD4 T cells in the portal tracts are infected and depleted, leaving T cells primarily in the centrilobular zone. However, the lack of a depletion of CD3+ liver T cells in the SIV-infected macaques suggests that CD8 T cells may be recruited. It will be interesting to understand what triggers them to traffic to the centrilobular zone and the consequences. It is notable that steatosis, inflammation, and fibrosis are often more severe in the liver centrilobular areas in adults with MAFLD.

HIV-associated liver disease is understudied due to the difficulty in collecting liver biopsies from people, resulting in limited data regarding liver pathologies in HIV-infected patients. Obesity-driven MAFLD and alcohol-associated fatty liver disease, on the other hand, are well-characterized in animal studies as well as in humans. Fat deposition in obesity-related MAFLD is most often macrovesicular steatosis as the condition leads to the accumulation of triglycerides within cells that form large nucleus-displacing fat droplets [2,50]. By contrast, we found microvesicular steatosis in the SIV-infected macaques, a condition associated with mitochondrial dysfunction and impairment of the beta-oxidation of fatty acids concurrent with increased PPARA expression [2,50]. This observation suggests a role for oxidative stress in the liver during acute SIV infection.

Taken together, these metabolic, histological, and anatomic findings indicate dysfunction of the liver during SIV infection that was initiated early upon infection and evolved over time. The acute phase liver pathology was characterized by evidence for microvesicular steatosis that resolved at later time points. Liver pathology within the blood plasma during the acute phase was evident by the significantly higher concentrations of AST and cholesterol. In contrast, the chronic phase of the infection was associated with sinusoidal dilatation, indicating compromised blood flow through the liver. The differential liver pathologies associated with the acute and chronic phases of infection provide insights regarding how the timing of therapeutic interventions could benefit liver health in people living with HIV. Future studies will need to evaluate the impact of antiretroviral drugs on the liver alone and in the context of SIV infection for a comprehensive understanding of liver pathology during HIV infection in the era of combined antiretroviral therapy.

## Data Availability

All data included in the manuscript are provided within the manuscript and its Supplementary Materials.

## Supplementary Materials

The following supporting information can be downloaded at: https://www.mdpi.com/article/10.3390/v16020296/s1, Table S1: Metabolic profiling in SIV-infected and SIV-naïve macaques; Table S2: Histological findings from SIV-infected and SIV-naïve macaques.
