# Supplementary material for: SIV Infection Is Associated with Transient Acute-Phase Steatosis in Hepatocytes In Vivo
Source: Viruses. 2024 Feb 15;16(2):296. doi: 10.3390/v16020296 (PMC10892327; doi:10.3390/v16020296)
Supplement: Supplementary file 1 [file viruses-16-00296-s001.zip › Supplementary Table S1.pdf]

Supplementary Table S1. Metabolic profiling in SIV-infected and SIV-naïve macaques.

Treatment Group	Animal ID	Time Point	AST	ALT	Chol.
**SIV-naive**	RM110	Wk -4	24	71	146
		Wk 2	34	175	147
		Wk 6	33	113	163
		Wk 16	23	85	168
		Nx	31	112	160
	RM111	Wk -4	29	38	106
		Wk 2	26	32	124
		Wk 6	27	37	120
		Wk 16	39	30	127
		Nx	43	33	124
	RM112	Wk -4	38	35	125
		Wk 2	40	48	141
		Wk 6	40	58	148
		Wk 16	46	111	116
		Nx	39	58	99
	RM113	Wk -4	34	29	110
		Wk 2	28	24	103
		Wk 6	35	34	101
		Wk 16	36	28	108
		Nx	52	41	122
	RM114	Wk -4	32	30	135
		Wk 2	40	20	128
		Wk 6	35	22	139
		Wk 20	40	40	160
		Nx	42	32	138
	RM115	Wk -4	36	28	130
		Wk 2	47	29	109
		Wk 6	59	33	114
		Wk 20	39	25	121
		Nx	40	27	153
	RM116	Wk -4	46	32	140
		Wk 2	47	32	141
		Wk 6	59	32	142
		Wk 20	40	32	131
		Nx	52	30	130
	RM117	Wk -4	34	27	145
		Wk 2	38	27	129
		Wk 6	49	32	150
		Wk 20	32	24	141
		Nx	28	29	179
**SIV-infected**	RM101	Wk -4	39	34	122
		Wk 2	67	40	183
		Wk 6	55	52	164
		Wk 16	n/a	n/a	n/a
		Nx	29	40	148
	RM102	Wk -4	62	47	92
		Wk 2	56	60	122
		Wk 6	33	35	109
		Wk 16	31	33	106
		Nx	33	39	114
	RM103	Wk -4	30	28	138
		Wk 2	41	27	213
		Wk 6	25	23	168
		Wk 16	23	21	149
		Nx	26	29	154
	RM104	Wk -4	26	31	111
		Wk 2	91	104	137
		Wk 6	25	39	128

		Wk 16	n/a	n/a	n/a
		Nx	n/a	32	n/a
	RM105	Wk -4	51	28	173
		Wk 2	136	31	146
		Wk 6	48	18	136
		Wk 20	41	25	163
		Nx	135	18	111
	RM106	Wk -4	29	31	100
		Wk 2	110	59	122
		Wk 6	31	24	96
		Wk 20	28	17	77
		Nx	35	26	104
	RM107	Wk -4	23	19	149
		Wk 2	68	41	196
		Wk 6	23	19	172
		Wk 20	19	19	169
		Nx	37	16	152
	RM108	Wk -4	24	17	149
		Wk 2	76	31	173
		Wk 6	30	15	178
		Wk 20	24	20	168
		Nx	41	26	153
	RM109	Wk -4	38	32	158
		Wk 2	120	48	160
		Wk 6	59	22	130
		Wk 20	n/a	n/a	n/a
		Nx	n/a	25	n/a

AST – aspartate aminotransferase; ALT – alanine aminotransferase; Chol – cholesterol; n/a – not available
