# Supplementary material for: SIV Infection Is Associated with Transient Acute-Phase Steatosis in Hepatocytes In Vivo
Source: Viruses. 2024 Feb 15;16(2):296. doi: 10.3390/v16020296 (PMC10892327; doi:10.3390/v16020296)
Supplement: Supplementary file 1 [file viruses-16-00296-s001.zip › Supplementary Table S2.pdf]

Supplementary Table S2. Histological findings from SIV infected and SIV naïve macaques.

Treatment Group	Animal ID	Time Point	Findings
**SIV-naive**	RM110	Wk 2	Macrovesicular steatosis 5%
		Wk 6	***Macrovesicular steatosis 15%***
		Wk 16	Macrovesicular steatosis 5%, ***single focus of lobular inflammation***
		Nx	Macrovesicular steatosis 5%, ***focal lobular inflammation***
	RM111	Wk 2	Macrovesicular steatosis 5%, ***sinusoidal dilatation***
		Wk 6	***Macrovesicular steatosis 15%***
		Wk 16	Macrovesicular steatosis 2%
		Nx	Macrovesicular steatosis 5%
	RM112	Wk 2	Macrovesicular steatosis 5%
		Wk 6	***Macrovesicular steatosis 15%***
		Wk 16	Macrovesicular steatosis 5%
		Nx	Macrovesicular steatosis 5%, ***CV fibrosis, scattered portal hemosiderin-laden macrophages and iron deposits in pan-lobular distribution***
	RM113	Wk 2	Macrovesicular steatosis 2-5%
		Wk 6	***Macrovesicular steatosis 5%, mild portal inflammation***
		Wk 16	Macrovesicular steatosis 2%, ***sinusoidal dilatation***
		Nx	Macrovesicular steatosis 5%
	RM114	Wk 2	Macrovesicular steatosis 5%, ***sinusoidal dilatation***
		Wk 6	Macrovesicular steatosis <2%
		Wk 20	Macrovesicular steatosis 2%
		Nx	Macrovesicular steatosis <5%, ***focal minimal portal and focal moderate central venulitis***
	RM115	Wk 2	***Macrovesicular steatosis 20%***
		Wk 6	***Macrovesicular steatosis 15%***
		Wk 20	***Macrovesicular steatosis 10%***
		Nx	***Mild sinusoidal dilatation, focal mild lobular inflammation***
	RM116	Wk 2	***Macrovesicular steatosis 10%***
		Wk 6	Macrovesicular steatosis 5%
		Wk 20	***Macrovesicular steatosis 15%, focal lobular inflammation***
		Nx	***Focal minimal portal inflammation, focal mild lobular inflammation***
	RM117	Wk 2	Macrovesicular steatosis 5%, **microvesicular steatosis 5%**
		Wk 6	Macrovesicular steatosis <2%
		Wk 20	***Macrovesicular steatosis 10%***
		Nx	***Mild sinusoidal dilatation, focal mild portal and lobular inflammation***
**SIV-infected**	RM101	Wk 2	***Microvesicular steatosis 80%, macrovesicular steatosis 10%***
		Wk 6	***Macrovesicular steatosis 10%***
		Wk 16	***Macrovesicular steatosis 15%***
		Nx	Macrovesicular steatosis 5%, ***sinusoidal dilatation***
	RM102	Wk 2	Macrovesicular steatosis 5%
		Wk 6	Macrovesicular steatosis 5%
		Wk 16	Macrovesicular steatosis 2%
		Nx	Macrovesicular steatosis 5%, ***sinusoidal dilatation***
	RM103	Wk 2	**Microvesicular steatosis 2%**
		Wk 6	Macrovesicular steatosis 5%
		Wk 16	Macrovesicular steatosis <1%
		Nx	Macrovesicular steatosis 5%, ***sinusoidal dilatation, moderate portal inflammation, portal expansion***
	RM104	Wk 2	Macrovesicular steatosis 5%, ***microvesicular steatosis 80%***
		Wk 6	***Macrovesicular steatosis 10%***
		Wk 16	Macrovesicular steatosis 2%
		Nx	Macrovesicular steatosis 5%, microvesicular steatosis <5%, ***mild sinusoidal dilatation***
	RM105	Wk 2	Macrovesicular steatosis 2%, ***microvesicular steatosis 40%***
		Wk 6	Macrovesicular steatosis 10%, ***focal mild portal inflammation***
		Wk 20	Macrovesicular steatosis 5%
		Nx	***Mild to moderate portal inflammation and venulitis***
	RM106	Wk 2	Macrovesicular steatosis 5%, ***microvesicular steatosis 60%, scattered apoptotic hepatocytes***

		Wk 6	Macrovesicular steatosis <2%, ***mild portal inflammation***
		Wk 20	Macrovesicular steatosis 5%, ***lobular inflammation, scattered apoptotic hepatocytes***
		Nx	Macrovesicular steatosis 5%, ***focal mild lobular inflammation***
	RM107	Wk 2	Macrovesicular steatosis 2%, ***microvesicular steatosis 30%***
		Wk 6	Macrovesicular steatosis 5%
		Wk 20	Macrovesicular steatosis 5%
		Nx	***Mild to moderate sinusoidal dilatation, mild portal and central venulitis***
	RM108	Wk 2	Macrovesicular steatosis 2%, ***microvesicular steatosis 70%***
		Wk 6	Macrovesicular steatosis <2%
		Wk 20	Macrovesicular steatosis 2%
		Nx	***Moderate sinusoidal dilatation, mild portal inflammation***
	RM109	Wk 2	Macrovesicular steatosis <2%
		Wk 6	Macrovesicular steatosis <2%, ***single focus of lobular inflammation***
		Nx Wk 9	***Mild sinusoidal dilatation, single focus of perivenular inflammation***
